# Measuring the Optical Properties of Highly Diffuse Materials

**DOI:** 10.3390/s23156853

**Published:** 2023-08-01

**Authors:** Mathieu Nguyen, Jean-Baptiste Thomas, Ivar Farup

**Affiliations:** 1 Department of Computer Science, Norwegian University of Science and Technology (NTNU), 2815 Gjøvik, Norway; 2 Imagerie et Vision Artificielle (ImVIA) Laboratory, Department IEM (Informatique, Électronique, Mécanique), Université de Bourgogne, 21000 Dijon, France

**Keywords:** material appearance, BSSRDF, inversion model, imaging device, absorption coefficient, reduced scattering coefficient

## Abstract

Measuring the optical properties of highly diffuse materials is a challenge as it could be related to the white colour or an oversaturation of pixels in the acquisition system. We used a spatially resolved method and adapted a nonlinear trust-region algorithm to the fit Farrell diffusion theory model. We established an inversion method to estimate two optical properties of a material through a single reflectance measurement: the absorption and the reduced scattering coefficient. We demonstrate the validity of our method by comparing results obtained on milk samples, with a good fitting and a retrieval of linear correlations with the fat content, given by $R^{2}$ scores over 0.94 with low *p*-values. The values of absorption coefficients retrieved vary between 1 × 10${}^{-3}$ and 8 × 10${}^{-3}$ mm${}^{-1}$, whilst the values of the scattering coefficients obtained from our method are between 3 and 8 mm${}^{-1}$ depending on the percentage of fat in the milk sample, and under the assumption of the anisotropy factor g>0.8. We also measured and analyzed the results on white paint and paper, although the paper results were difficult to relate to indicators. Thus, the method designed works for highly diffuse isotropic materials.

## 1. Introduction

Diffuse materials are known for redistributing light through phenomena such as subsurface scattering. These materials can be found in everyday objects such as light and displays [1], or food and liquids such as milk [2]. Being able to estimate the optical properties of a material such as the absorption or scattering coefficients can be useful for various applications: the diagnosis of diseases with the analysis of skin tissues [3,4,5,6], nondestructive quality control of fruits and vegetables [7,8,9], rendering of skin faces and other materials for the movie, gaming and virtual reality industries [10].

According to Tong et al. [11] in their study on modeling and rendering the subsurface scattering of quasi-homogeneous materials, two different approaches can be considered to describe the appearance. The first approach can be called the **object appearance representation**, and it focuses on capturing appearance features coupled to a specific geometry of an object. Therefore, these properties are intrinsic to the object considered and cannot be exported directly to a wider scope. It is practical to focus on the object appearance when it comes to assessing the quality of specific objects, or if the object geometry is original in itself. The second approach is the **material appearance representation**. It consists of studying the properties which define the appearance of the material regardless of the geometrical shape of the object it may compose. Then, they can define the material at an industrial production level. Features such as colour, texture, gloss, translucency, or transparency can be utilized to describe the appearance of a material [12]. These features are frequently correlated with optical properties of the material such as absorption, scattering, transmittance or reflectance at specific wavelengths. They represent how the light interacts with the material surface or how it propagates inside and through the medium. We aim to estimate the optical properties related to absorption and scattering of highly diffuse materials, such as milk, prone to scattering and subsurface scattering effects. Thus, in this work, we consider the material appearance over the object appearance representation.

Subsurface scattering is influencing the material appearance. Among their introduction of several concepts on reflectance properties, Nicodemus et al. [13] define the subsurface scattering phenomenon as diffuse reflections produced by multiple internal scattering in a material below the nominal reflecting surface. This effect occurs with diffuse reflectors and almost all natural reflecting surface materials. It highly depends on how the incident light beam penetrates into the medium before being absorbed or scattered back through the surface again. In most cases, the exit point for rays is different from the entry point in the material. We considered the assumption of having a semi-infinite material. Hereby, we consider materials that are large enough to not consider all boundary effects, and make it impossible for light to escape on the sides and to see through.

Scattering and absorption effects usually vary with the wavelength of the light considered [14], and can then be numerically characterized, respectively, by the scattering coefficient $\mu_{s}$ and the absorption coefficient $\mu_{a}$. Effects of scattering and absorption contribute to the material appearance and their absences in virtual rendering are noticeable. For example, they influence skin rendering for human faces due to the various layers under the skin and the concentration of hemoglobin [15]. As a consequence, being able to estimate the values of the scattering and absorption coefficients of a material can be an asset to improve its rendering, assuming an adequate model is chosen.

The focus of this work is on the use of the diffusion theory model [16] coupled with an adapted implementation of a nonlinear least-square inversion method to estimate the absorption and scattering coefficients linked to the optical properties of highly diffuse materials. Two surveys from the literature are useful for this study. Guarnera et al. [10] present an overview on how to capture material information related to its appearance, how to model it and apply it for virtual reality usages. Frisvad et al. [17] reviewed several models and ways to measure optical properties of translucent materials and give a clear definition of the design of an inversion model. Both surveys introduce the notion of the appearance model for computer graphics application, but this can also be used for material study. Appearance models can consider two aspects [11]: object appearance and material appearance. Optical properties related to material appearance are commonly supported by models which explain the propagation of light inside a medium such as the Kubelka–Munk model [18], the four-flux model [19], the Inverse Adding Doubling method [20,21] or the diffusion model [16], or by functions such as the BRDF (Bidirectional Reflectance Distribution Function) and BSSRDF (Bidirectional Subsurface Scattering Reflectance FUnction) [13] to describe the reflectance of light by a material. Two optical properties can be identified related to material appearance and subsurface scattering [3,22]: the absorption coefficient and the scattering coefficient. They can be retrieved by performing acquisitions of BSSRDF using various setups [23,24,25], or by measuring the attenuation of light in the medium and solving the inversion problem from the collected data [8,9,14,26].

The radiative transfer equation is a model describing the behaviour of light scattering and absorption in a material. In the case of material with dominant scattering, a diffusion model may be more adapted. Such a model has been widely used in the literature for various applications. Following the initial use of Farrell et al. [16], Kienle et al. performed measurements of optical absorption and scattering coefficients of biological tissues by using a noninvasive approach they developed [26]. They also pursued this work by focusing on the measurements of absorption and scattering coefficients in the case of semi-infinite turbid media in which they evaluated their method with the use of time and frequency domain acquisition techniques and Monte Carlo simulations [27,28]. Another area for which the diffusion model is being utilized is in food quality control. Qin et al. applied a hyperspectral imaging method to liquids such as milk and juices [7], or fruits and vegetables [8] to obtain the absorption and reduced scattering parameters of the material studied. A similar technique was followed by Hu et al. for studying fruits with visible and near-infrared imaging devices [9]. Stam worked on the diffuse approximation itself and reviewed several numerical solutions to solve the radiative transfer equation using the approximation [29]. Finally, this model was extended to the area of computer graphics by Jensen et al. [30] with the implementation of the dipole model adapted to the representation of the BSSRDF of materials, which is mainly used in the rendering literature [31].

Farrell et al. [16] studied the diffuse reflectance $R_{d}$ of an infinite small vertical light source upon the surface of a semi-infinite turbid medium as a function of the radial distance between the source and the detector. It allowed them to study the optical parameters of the medium such as the absorption coefficient $\mu_{a}$, the reduced scattering coefficient $\mu_{s}^{\prime }$, and the relative refractive index η. Equation (1) gives the diffuse reflectance,

(1) $$R_{d}\left(r\right)=\frac{\alpha }{4\pi \mu_{t}}[(\mu_{\mathrm{eff}}+\frac{1}{r_{1}})\frac{e^{-\mu_{\mathrm{eff}}r_{1}}}{r_{1}^{2}}+(1+\frac{4A}{3})(\mu_{\mathrm{eff}}+\frac{1}{r_{2}})\frac{e^{-\mu_{\mathrm{eff}}r_{2}}}{r_{2}^{2}}]$$

where *r* is the distance between the entry point of the light in the material and the detected exit point, α is the reduced albedo defined by $\alpha =\frac{\mu_{s}^{\prime }}{\mu_{a}+\mu_{s}^{\prime }}$, $\mu_{\mathrm{eff}}$ is the effective attenuation coefficient defined by $\mu_{\mathrm{eff}}=\sqrt{3\mu_{a}\left(\mu_{a}+\mu_{s}^{\prime }\right)}$, and $\mu_{t}$ is the extinction coefficient defined by $\mu_{t}=\mu_{a}+\mu_{s}^{\prime }$. $r_{1}$ and $r_{2}$ are distances of the observed point at the surface, respectively, from the real and virtual source (dipole approximation) such as

(2) $$r_{1}=\sqrt{r^{2}+(\frac{1}{\mu_{t}}{)}^{2}}$$

(3) $$r_{2}=\sqrt{r^{2}+[(1+\frac{4A}{3})\frac{1}{\mu_{t}}{]}^{2}}$$

In Equations (1) and (3), the parameter *A* is an internal reflection coefficient and it can be derived following the equations proposed by Donner et al. [31]

(4) $$A=\frac{1+F_{d}}{1-F_{d}}$$

with $F_{d}$ the diffuse Fresnel reflectance defined by

(5) $$F_{d}=\left.\left\{\begin{array}{cc} -0.4299+\frac{0.7099}{\eta }-\frac{0.3319}{\eta^{2}}+\frac{0.0636}{\eta^{3}} & \eta <1\\ -\frac{1.4399}{\eta^{2}}+\frac{0.7099}{\eta }+0.6681+0.0636\eta & \eta >1 \end{array}\right.\right.$$

The relative refractive index η is defined as the ratio of refractive indices between the two media considered at the interface (air and the material in our case). Most of the materials used are assumed to have a constant refractive index to avoid having a complex model. Therefore, once the reflectance profile of a material is known, it is possible to have estimates of the coefficients $\mu_{a}$ and $\mu_{s}^{\prime }$ by performing an inversion of Equation (1). Assumptions of this model used in this study are that the material is semi-infinite, i.e., both its thickness and its lateral dimensions are infinite, and the material is highly scattering.

We selected the diffuse approximation as the light model to fit the measured data, and the spatially resolved (SR) method as the way to perform the acquisition. The SR method has often been utilized in various setups for measuring optical properties. Nichols et al. [32] used a white light source to acquire reflectance from biological tissues through optical fibers close to the sample. Doornbos et al. [14] performed a similar experiment with optical fibers but they were able to have a larger spectrum for the light source, thus introducing a wavelength dependency model for the scattering coefficient. Qin et al. [7] made use of hyperspectral imaging for studying optical properties of fruits and vegetables. Their setup was composed of a CCD camera coupled with an imaging spectrograph and a zoom lens to get an image while the samples were illuminated with a collimated light. Although this setup allows hyperspectral imaging and a better understanding of the behaviour of the scattering and absorption properties based on the wavelength of the light, its use is limited to very constrained conditions. Thus, we selected an industrial imaging-based device called the ©Dia-Stron TLS850 to perform the acquisitions on materials. Its ergonomics allows for both indoor and outdoor measurements if opportunities are presented. We chose to test our inversion method with the diffusion model on highly diffuse materials with liquid and solid physical states. Some of the highly diffuse materials can be associated with the colour white (not the case of skin), and thus have strong reflective properties. They represent a challenge for several imaging techniques due to the saturation of sensors or other calibration issues, which makes them an interesting target for studying.

The material we have selected to verify and prove our inversion method is cow milk. Milk is a diffuse material with high scattering properties due to the casein and fat particles which compose it [33]. The fat percentage of milk usually characterizes its properties [34], and a linear relationship with the absorption and scattering coefficients has been shown in several studies [35,36]. Therefore, measuring the optical coefficients of milk and their linear relationship to the fat content of milk is an ideal scenario to validate our acquisition and inversion method. Furthermore, there have been other recent studies using spectroscopy methods to analyze the composition of milk impacting the quality of dairy products [37,38].

The Section 1 deals in-depth with the measurement protocol employed, the device used and the inversion method put in place to solve the problem. A Section 2 sheds light on the validation of the method using measurements on milk samples with various fat content, estimations of absorption and reduced scattering parameters, and retrieval of linear correlation between the fat content of the milk and its optical properties. In a Section 3 the results results are presented on other highly diffuse materials such as paper substrates and tainted water with white paint pigments, before concluding on the validity of the method and its limitations.

## 2. Method

### 2.1. Acquisition

**Figure 1:**
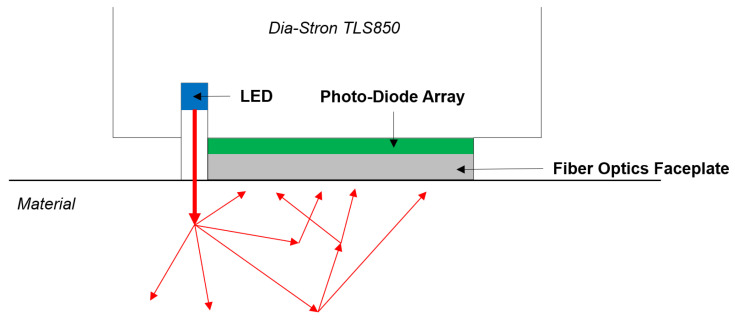
Schematic of the process operated by the TLS850. The LED sends light vertically towards the surface of the material, then the light is scattered in it. The photodiode array collects the light coming out of the material at various distances from the entry point in the material.

We selected a translucency meter device called the ©Dia-Stron TLS850. It is a hand device composed of 3 LEDs for RGB channels and a 20 mm photodiode array to detect signal of re-emitted light from the material sampled. Figure 1 is a schematic representation of what the device does when the acquisition is started, and it illustrates the spatially resolved method previously mentioned. The design is practical and allows a wide variety of uses, whether it is the acquisition conditions (indoor or outdoor) or the physical state of the material (solid or liquid are possible). By reproducing the spatially resolved method, it allows the user to study the attenuated intensity along the distance from the light source for red, green and blue channels. The peak wavelengths are, respectively, at 630 nm, 525 nm, and 472 nm according to the manufacturer. There is also the possibility to fine tune the LED control to have separate or simultaneous use of the three channels. The sensor of the device is an NMOS linear image sensor type composed of 512 pixels whose pixel pitch is 25 µm and pixel height is 2.5 mm. The spectral response range of the sensor varies from 200 nm to 1000 nm with a peak sensitivity at 600 nm. Furthermore, the photodiode typical dark current is at 0.1 pA with a maximum of 0.3 pA, and its saturation charge is at 25 pC. The combination of a low dark current and a high saturation charge allows the photodiode to reach a long integration time and a wide dynamic range at ambient temperature. The total acquisition time for one sample with the 3 LEDs operating in sequence varies between 8 and 10 s, which averages up to 3 s per LED.

### 2.2. Inversion

In their review, Frisvad et al. [17] presented several models and ways to estimate the optical properties of translucent materials. Even though our study focuses on the optical properties of highly diffuse materials, their description of the forward and especially inverse model is valuable. When designing an the inverse model, some steps must be validated. The first aspect is to define the type of data measured, then a model for light scattering distribution must be chosen. Afterwards, an objective function is to be selected to give the best match possible between the model and the measurements. Finally, an optimization method is applied to minimize the objective function. In our case, reflectance data profiles are acquired, the diffusion model is chosen as the light transfer model, and we have selected a trust-region nonlinear optimization method to optimize the sum of squares of residuals between the data and the model.

**Figure 2:**
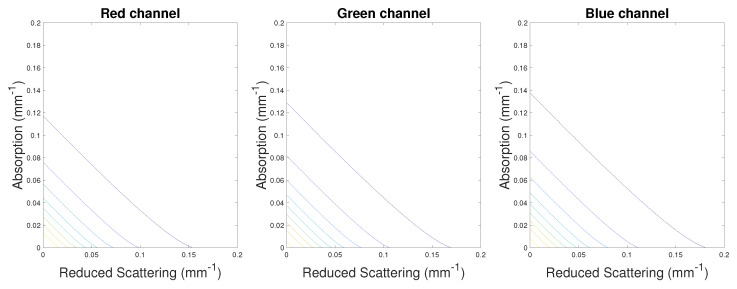
Contour plot of an arbitrary study of the cost function. The lines show the potential ($\mu_{a},\mu_{s}^{\prime }$) solutions minimizing the cost function. An ideal solution would be to find an ellipse made of these contours, which is not the case in this study.

As stated by Kienle et al. [26], there could be an infinite number of solutions for $\mu_{a}$ and $\mu_{s}^{\prime }$ if there are no constraints in the optimization process. In their paper, Jensen et al. [30] used the diffuse equation as reported in Equation (1). For their optimization, they measured the total reflectance, which is the integration of $R_{d}$ over the whole surface considered. Therefore, they added this constraint to obtain estimates of $\mu_{a}$ and $\mu_{s}^{\prime }$. Nonetheless, acquiring the total reflectance may not always be possible, which is the case in this work. For this case, we selected milk data from our dataset and we assigned values in the range [0, 0.2] mm${}^{-1}$ to absorption and reduced scattering coefficients. For each of these values, we computed the sum of square residuals between the data and the model we wanted to fit, and we plotted the contour lines for each channel R, G, and B. As seen in Figure 2, for each channel, it is not possible to find an area for which a set $\left(\mu_{a},\mu_{s}^{\prime }\right)$ minimizes the cost function chosen. There seems to be an infinite number of solutions judging by the straight lines plotted. This result strongly indicates there is a need to refine the optimization method to add more constraints in order to find estimates for $\mu_{a}$ and $\mu_{s}^{\prime }$ which would minimize the cost function.

Farrell et al. [39] studied the influence of the optical parameters $\mu_{\mathrm{eff}}$ and $\mu_{t}$ on the shape of the reflectance. According to them, most of the information of those optical parameters can be found in the shape of the reflectance. Rather than looking directly at the reflectance, they focused on $\log|r^{2}R_{d}\left(r\right)|$ while fixing the value of one parameter and varying the other one in a Monte Carlo simulation. When $\mu_{\mathrm{eff}}$ is fixed, they observed a peak in the shape of the curve a few millimeters of the light source. The position of this peak changed depending on the value of $\mu_{t}$ chosen. Similarly, when $\mu_{\mathrm{eff}}$ varies while $\mu_{t}$ is fixed, when detected away from the source, the slope of the exponential curve increased or reduced. Then, they concluded that the two coefficients $\mu_{\mathrm{eff}}$ and $\mu_{t}$ could be estimated by analyzing the shapes of those curves, and therefore could deduce the value of the absorption $\mu_{a}$ and the reduced scattering $\mu_{s}^{\prime }$ coefficients.

To corroborate their conclusion, we investigated Equation (1) by doing asymptotic derivations in the case of r→0 and r→+∞. The derivations were performed on a normalized version of Equation (1) as the interest lies into the shape of the diffuse reflectance, and since it is not always possible to obtain the total reflectance for each sample, it is best to actually focus on the shape. Then, the coefficient outside the bracket in Equation (1) is discarded by the normalization process. After derivations, the following equations are obtained:

(6) $$R_{d}\left(r\right)\underset{r\to \;0}{=}\mu_{t}^{3}(1+\frac{1}{C^{2}})$$

(7) $$R_{d}\left(r\right)\underset{r\to \;+\infty }{=}\left(1+C\right)(\mu_{\mathrm{eff}}+\frac{1}{r})\frac{e^{-\mu_{\mathrm{eff}}r}}{r^{2}}$$

with $C=1+\frac{4A}{3}$. Using the result of Equation (6), the shape of the diffuse reflectance is mainly influenced by the parameter $\mu_{t}$ in the case of r→0, when the light is detected close to the source. Similarly, when the light is detected far away from the source, the diffuse reflectance can be approximated by the expression of Equation (7). The parameter $\mu_{\mathrm{eff}}$ is mainly shaping the curve of the diffuse reflectance with the exponential decrease. These results support the conclusion of Farrell et al. [39] but also the results from Bevilacqua et al. [40] where they stated that to obtain a unique determination of $\left(\mu_{a},\mu_{s}^{\prime }\right)$, it is required to perform at least two sets of measurements with one close to the source and one far from the source.

As a consequence, we decided to split our reflectance data curves into two parts to estimate differently the effective attenuation coefficient $\mu_{\mathrm{eff}}$ and the extinction coefficient $\mu_{t}$. We need to determine at which distance from the source the data can be split in two parts. To do so, we compute the gradient of $f\left(r\right)=r^{2}R\left(r\right)$. According to the observations of Farrell et al. [39], the peak of this function *f* could be used as a separation for our data. The use of the gradient helps to find the position of the peak by looking at the change of sign of the gradient, which symbolises a change of variation in the function. As such, the split of the data would be adapted to each type of data for each channel R, G, and B acquired. This method gives estimated values for $\mu_{t}$ and $\mu_{\mathrm{eff}}$, and by using their analytical expressions, both absorption and reduced scattering coefficients can be deduced with

(8) $$\mu_{a}=\frac{\mu_{\mathrm{eff}}^{2}}{3\mu_{t}}$$

(9) $$\mu_{s}^{\prime }=\mu_{t}-\frac{\mu_{\mathrm{eff}}^{2}}{3\mu_{t}}$$

Therefore, for each part of the curve, we performed a trust-region nonlinear least-square optimization [7] to get estimates of $\mu_{t}$ and $\mu_{\mathrm{eff}}$ and then compute both optical parameters using Equations (8) and (9).

## 3. Results and Validation on Milk

We assembled two datasets of milk samples to perform our measurements. The first dataset is referred as the Q-dataset and is composed of four types of milk samples from the brand ©Q, a Norwegian milk company whose bottles can be bought in supermarkets. The fat concentration in these milk samples can be found at 0.1%, 0.5%, and 1% to 4%. The second dataset is also composed from various samples of milk, ranging from skim milk (0.1% of fat), light milk (0.5%, 0.7%, 1% and 1.2%), to whole milk (3.5% and 4%). Since the samples originate from different brands and some variations in the making and dilution processes may have been introduced by the companies, this dataset was named the mix-dataset.

For both datasets, we followed a similar protocol of measurement. A cylindrical plastic glass was filled to its maximal volume capacity with milk, which represented nearly one liter. The glass has a diameter of 9 cm and a height of 16 cm. The ©Dia-Stron was attached to a tripod with an adjustable height. Therefore, we were able to set up the device so that the sensor was in contact with the milk but not entirely immersed into it. All measurements were performed in a dark room to avoid noise and perturbations from other light sources. The value for the refractive index of milk is assumed to be 1.347 [36]. The results for each dataset are discussed in the following paragraphs.

### 3.1. Q-Dataset Results

We first detail the results of the experiment conducted on the Q-dataset composed of milk samples from the Norwegian ©Q brand. Table 1 shows the values of absorption coefficient $\mu_{a}$ and reduced scattering coefficient $\mu_{s}^{\prime }$ estimated from the reflectance profiles using the inversion method described earlier. A first aspect to note is that $\mu_{s}^{\prime }$ values are larger than $\mu_{a}$ values by 2–3 orders of magnitudes, which validates the diffusion model utilized as one of its assumption is to have a medium with dominant scattering over absorption. A second positive aspect of the results in Table 1 is the increase in $\mu_{a}$ and $\mu_{s}^{\prime }$ values with the decrease in the wavelength for each channel. As pointed out by Doornbos et al. [14], a power-law relationship can be established between the reduced scattering coefficient and wavelengths. One could suggest to verify such a relationship by using the estimated values of Table 1, but only having three discrete values for three distinct wavelengths (472 nm for the blue channel, 525 nm for the green, and 630 nm for the red) may not be enough to draw conclusions. Moreover, the wavelengths previously given are the peak wavelengths of the LEDs power and it may not be as precise as a monochromator. Since our focus is not specifically on the spectral aspect, the lack of precision in spectral resolution is acceptable.

**Figure 3:**
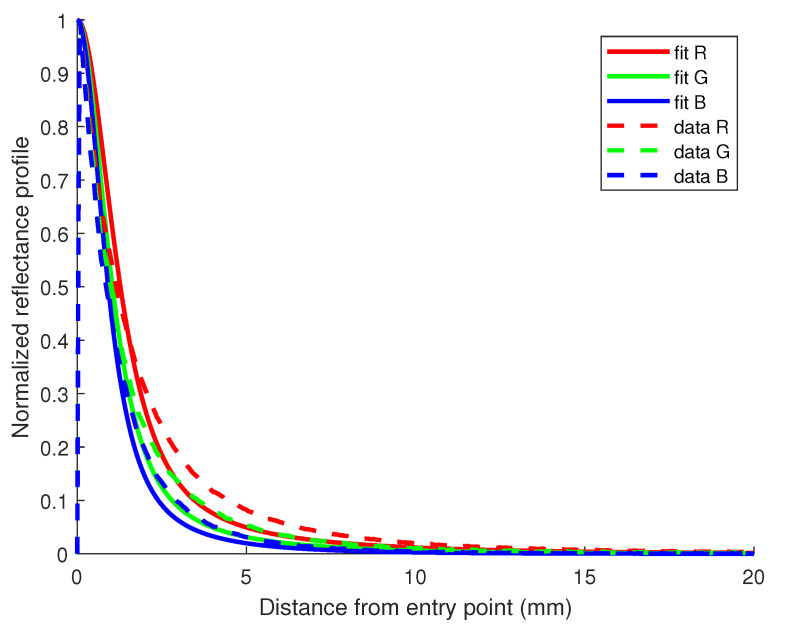
Reflectance profile for the milk sample containing 0.5% of fat in the Q-dataset. $R^{2}$ values for the fit of R, G, and B channels are all 0.90.

Another way of evaluating the quality of the estimates obtained is to plot the fit model and compare it to the data, as shown in Figure 3, where we display a plot with reflectance profiles acquired with the device and their fit model for each R, G, and B channel in the case of the milk sample containing 0.5% of fat concentration. The quality of the fit is evaluated using $R^{2}$ values computed as residuals between the reflectance profiles and Equation (1) of the diffusion model using the estimated values of absorption $\mu_{a}$ and reduced scattering $\mu_{s}^{\prime }$ coefficients from Table 1. We are dealing with normalized reflectance profiles when processing the comparison, since most of the information about these two optical properties is mainly contained in the shape of the reflectance curve [39]. Results for other fat concentrations show similar $R^{2}$ values, stating the estimated values provide a good fit to the model proposed.

### 3.2. Mix-Dataset Results

Similarly to the Q-dataset results, this section highlights the results of the mix-dataset which is composed of milk samples coming from various brands. Acquisitions were performed identically to the Q-dataset but are separated by a month in time. In Table 2, the results of the inverse algorithm are registered for estimating the optical properties following the acquisitions of reflectance profiles with the TLS850 device. Once again, one can notice that $\mu_{s}^{\prime }$ values are larger than $\mu_{a}$ values, which is expected in the case of a highly scattering material. Actually, as mentioned by Leyre et al. [2], the fact that scattering is more dominant in such a diffuse material as milk lowers the accuracy which can be reached for the estimate of the absorption coefficient. Furthermore, absorption is very low for milk.

**Figure 4:**
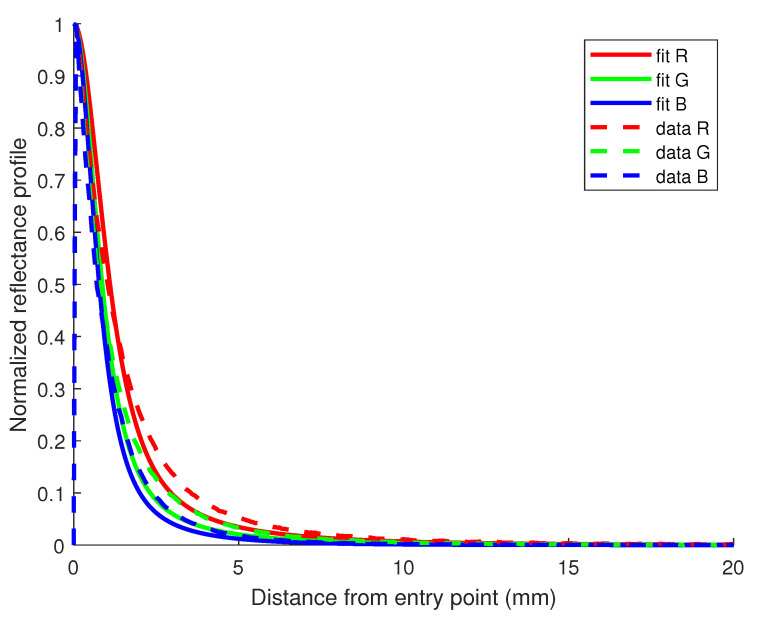
Reflectance profile for the milk sample containing 1.2% fat in the mix-dataset. $R^{2}$ values for the fit of R, G, and B channels are, respectively, 0.91, 0.90 and 0.89.

It is also possible to judge the quality of the fit of the model with the data using $R^{2}$ values supported with the example of Figure 4 for the case of the sample with a 1.2% fat content.

### 3.3. Correlation with the Fat Content

**Figure 5:**
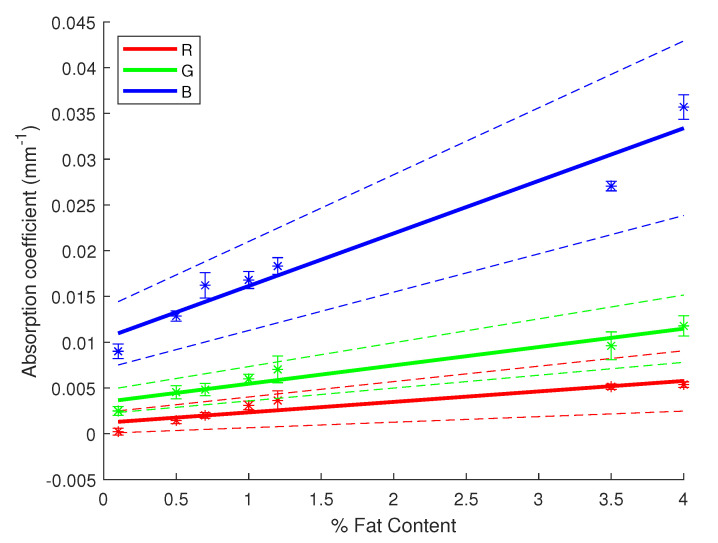
Linear regression for the absorption coefficient of milk versus the fat content with 95% confidence intervals (dashed lines). Star symbols (*) represent the value of the coefficient with the uncertainty bar.

**Figure 6:**
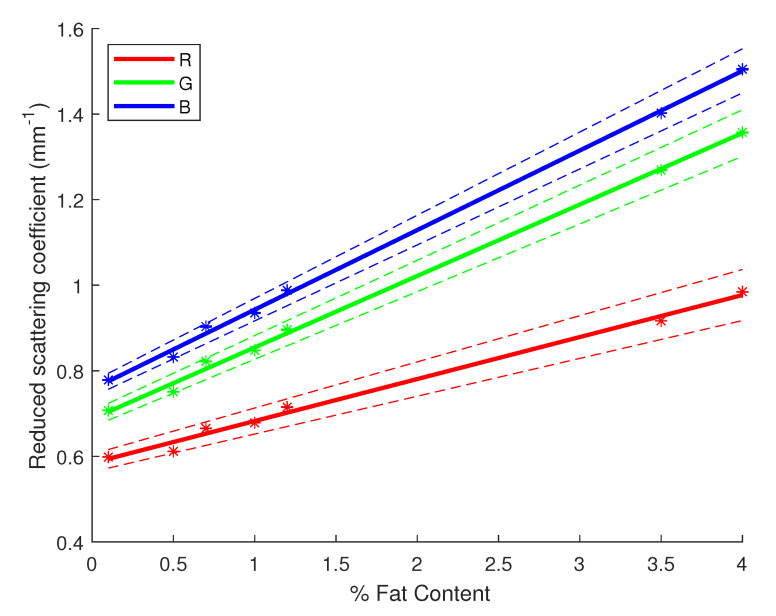
Linear regression for the reduced scattering coefficient of milk versus the fat content with 95% confidence intervals (dashed lines). Star symbols (*) represent the value of the coefficient with the uncertainty bar.

Another way of assessing the performance of our inversion method is by retrieving the linear relationship between the fat content of milk and the absorption and reduced scattering coefficients. For each milk sample, with a different fat percentage, 10 signals were acquired before computing the estimated coefficients for each of them. Thus, we can compute some statistics on the estimated values while verifying their linear relationship with the fat content of milk. Figure 5 and Figure 6 display the results of these statistical studies regarding the linear correlation with the fat content. The linear regression was performed with the average estimates of $\mu_{a}$ and $\mu_{s}^{\prime }$ for each sample of milk, then error bars were plotted using the 95% confidence interval ${\mathrm{CI}}_{95\%}=\pm 1.96\frac{\sigma }{\sqrt{N}}$ with σ being the standard deviation of the estimates for each sample and *N* the number of acquisitions per sample, hereby 10. In Figure 5 and Figure 6, the linear regressions (solid lines) for each channel were evaluated by computing confidence intervals at 95% (dashed lines), as well as $R^{2}$ and *p*-values, as shown in Table 3. One can notice the obtained results validate a correlation between the fat content of milk and its absorption and scattering properties. Indeed, $R^{2}$ values are very high and close or equal to 1 while the *p*-values are very low, giving more weight to the probability of an existing linear correlation. Furthermore, in Figure 6, one can observe the regression lines for each channel have almost a similar slope since they are nearly parallel (mostly the case for the blue and green channel). It shows the scattering of milk mainly varies in magnitude depending on the light’s wavelength, but the variation in the fat content remains almost constant across the visible spectrum. In previous studies, it has been shown that fat particles inside the milk play a major role with the interaction of light in the milk. Being able to retrieve the correlation with our inversion method validates its use.

### 3.4. Comparison with the Literature

There exist several studies in the literature using milk as a test material for estimating absorption and reduced scattering coefficients [7,33,36], which is one of the reasons why this highly diffuse material was chosen to test our method. Thus, it is possible to compare the results obtained from our inversion method to the estimates found in the literature. Some of the studies such, as Jensen et al. [30], do not provide estimates for the reduced scattering coefficient $\mu_{s}^{\prime }$, but rather consider the scattering coefficient $\mu_{s}$ of the material. These two optical parameters are related together by the following equation:

(10) $$\mu_{s}=\frac{\mu_{s}^{\prime }}{1-g}$$

with *g* being the average of the cosine of the scattering angle of the material, also called the anisotropy factor. In the case of a material with high forward scattering properties, *g* would get a value close to 1, g=0 would mean an isotropic material and g=−1 would be for a material with strong backward scattering. As a consequence, by using Equation (10) for a high scattering material such as milk, (1−g) could be a very small value. Then, the $\mu_{s}$ value obtained would be an increased value of the estimated $\mu_{s}^{\prime }$ by several orders of magnitude. Assuming g>0.8, one would need to multiply our values of reduced scattering by a factor 5 to 10 to obtain scattering coefficient values to compare with the literature. One should be aware of this difference in the definition of the two parameters when comparing our results to others in the literature.

Nevertheless, we are able to compare our values obtained from the milk samples with previous references from the state of the art. Using an integrating sphere to perform their measurements, Stocker et al. [33] conducted a thorough study of the optical properties of milk. Their values for both absorption and reduced scattering coefficients were studied for a spectral purpose. Thus, when comparing their estimates to ours, one must only consider the visible range from 400 to 800 nm. The range of values they obtained for the scattering coefficient varies from 1 to 3 mm${}^{-1}$, and the range for the absorption coefficient is between 1 × 10${}^{-3}$ mm${}^{-1}$ and 1 × 10${}^{-2}$ mm${}^{-1}$. Their ranges match the ones we obtained from Table 1 and Table 2, even though we used different measurement methods. Focusing on other references with numerical values of the optical properties of milk, Qin et al. [7] also made use of a spatially resolved method with the diffusion approximation to get their estimates. Regardless, the ranges of their estimates are slightly superior to ours (between 0.5 and 2 cm${}^{-1}$ for $\mu_{a}$). Similarly, Abildgaard et al. [36] determined the diffusion center of incoming light in a material to obtain estimates of $\mu_{a}$ and $\mu_{s}^{\prime }$ using a different formula from Equations (8) and (9). Their results are also larger than ours (less than 1 mm${}^{-1}$ for $\mu_{a}$ values). The difference observed in the results could be due to the milk samples used for the experiment, since each study had their own different samples, or due to the measurement method utilized since they are different from one study to the other, ours included. Yet, as shown, the estimated results have similarities and overlaps. Therefore, one may conclude there is no method better than the others and that our inversion method is coherent.

### 3.5. Repeatability of Measurements

Another focus on the results for the milk material is their repeatability. It occurs that between the Q-dataset and the mix-dataset, there are common samples of milk containing the same fat content while measurements were conducted at separate instances of time. Thus, a comparison can be made for $\mu_{a}$ and $\mu_{s}^{\prime }$ values for the samples of 0.1%, 0.5%, 1%, and 4% of fat content. These results are reported in Table 4, computed as the difference between values for the Q-dataset and values for the mix-dataset and then divided by the Q-dataset values taken as the references. When first observing the relative difference for the reduced scattering coefficients, one can notice the resulted differences are low. It is a clear indication our inversion method performs well for repeated measurements regarding $\mu_{s}^{\prime }$ values, especially in the case of highly diffuse materials with high scattering properties. As mentioned earlier, the largest relative differences observed from Table 4 are for the absorption coefficient $\mu_{a}$ since these values are small from the beginning and thus more complicated to estimate. The consistency of repeated results for reduced scattering values tends to indicate our inversion method is operating accordingly.

### 3.6. Discussing the Inversion Method

One contribution of this paper is the inversion method. The process with the nonlinear fitting was changed as explained in the Section 2. Nonetheless, the algorithm procedure and the management of the reflectance data have their importance on the final estimates. For each sample of the materials considered, several records (between 10 to 15) were acquired each time. The inversion method for estimating the optical properties was implemented with two variations. The first variation considers all the records for a material sample. Then, the inversion algorithm is applied on each of them, giving individual ($\mu_{a},\mu_{s}^{\prime }$) values for each record. Afterwards, the final estimate of ($\mu_{a},\mu_{s}^{\prime }$) is the average of all the individual estimates. The second variation considers the average of all the records, which removes potential noise from the sensor, and then perform the inverse algorithm to estimate ($\mu_{a},\mu_{s}^{\prime }$) values. Therefore, the main difference between the two variations is when the average is performed, after or before the inversion, and on which quantities it is applied, the estimates from all the records or the records themselves.

We compared the two variations by using the data of the milk dataset and computing estimates of absorption and reduced scattering coefficients. Values reported in Table 1 and Table 2 are obtained using the second variation of the algorithm as previously mentioned. The relative difference between each value of ($\mu_{a},\mu_{s}^{\prime }$) for all milk samples from the two variations were then computed by taking the second variation as a reference. For this comparison, the criteria of the dataset (Q or mix) and the colour channel are discarded, i.e., we have 33 values of $\mu_{a}$ and 33 values of $\mu_{s}^{\prime }$ to be compared together for each variation. The relative difference obtained for absorption values is of −6.3% while the relative difference for reduced scattering values if of −0.3%. Overall, when combining both of them, we obtain a general relative difference of −3.3% between the two variations. Since the second variation is taken as the reference, a negative relative difference means the first variation provides slightly larger values than the second variation. As shown by the values, the highest differences are due to the estimates of $\mu_{a}$. Milk is a material with higher scattering properties than absorption, which leads to lower estimates of the absorption coefficient. These smaller values of $\mu_{a}$ are thus more challenging to estimate accurately and can generate large relative differences even though they remain small in absolute value compared to $\mu_{s}^{\prime }$ values. Therefore, its is not surprising to observe these relative differences of −6.3% for $\mu_{a}$ values and −0.3% for $\mu_{s}^{\prime }$ values. Both variations of the algorithm provide similar estimates of the reduced scattering coefficient. This comparison also gives good results regarding the robustness of the inversion method since close estimates are obtained from both variations.

Another point of discussion regarding our method would be the amount of light collected by the NMOS sensor of the measuring device. It is unsure if the light reflected at the surface of the material is entirely collected by the sensor, and most likely there are small losses. Nonetheless, since our inversion method focuses on the shape of the reflectance signal, we only need normalized data. Thus it is acceptable to not have the entirety of the light collected by the sensor, especially in the case of a single measurement setup such as ours, as it could be complicated to verify and ensure.

## 4. Other Highly Diffuse Materials

Our method was validated by using the milk material, but there are other highly diffuse materials on which one can utilize our method. Thus are presented in the following sections results of the estimated optical properties of white paper substrates and tainted water with white acrylic paint.

### 4.1. Optical Properties of Paper

For this experiment, we considered six categories of A4 white paper sheets by selecting six different paper weights: 80 g/m${}^{2}$, 100 g/m${}^{2}$, 120 g/m${}^{2}$, 160 g/m${}^{2}$, 200 g/m${}^{2}$, and 250 g/m${}^{2}$. As prescribed by the assumptions of the diffusion model, the material considered should have a semi-infinite thickness and width. To achieve that, for each samples of paper considered, several sheets were stacked together into reams. 500 A4 sheets were stacked for 80 and 100 g/m${}^{2}$ while 250 A4 sheets for the other weights, which corresponds to a thickness of 5.5 cm for 80 g/m${}^{2}$, 5.3 cm for 100 g/m${}^{2}$, 3.2 cm for 120 g/m${}^{2}$, 4.3 cm for 160 g/m${}^{2}$, 5.0 cm for 200 g/m${}^{2}$ and 6.3 cm for 250 g/m${}^{2}$. The sensor device was also put on top of the stack in the middle area of the sheet to ensure a semi-infinity in planar directions and avoid having light escaping on the edges. Since all the paper sheets were compactly stacked together, we assume the air interface between each sheet is too thin to be considered, and then it allows us to utilize the diffusion model. Further information required for our inversion method is the knowledge of the real part of the refractive index for the material considered. In the case of white paper, we used the study of Fabritius et al. [41] to obtain the value η=1.557.

Due to the anisotropic nature of the surface of paper and its directionality, we divided our sets of acquisitions by changing the position of the rectangular sensor in relation to the orientation of the stack. Therefore, we defined two datasets for our measurements. One is called the Vertical dataset and it corresponds to the sensor being orthogonal to the orientation of the paper stack. For example, if the stack is oriented into portrait, then the sensor shall be in landscape. The second dataset is labelled as the Horizontal dataset to specify the case in which the sensor is in the same orientation as the sheets. Results are presented in the following paragraphs.

#### 4.1.1. Vertical Dataset Results

Using our implemented inversion method coupled with the diffusion model, we were able to fit our data and obtain estimates of the absorption and reduced scattering coefficients for the paper sheets with the Vertical configurations. Results of these estimates for each class of paper and each R, G, and B channel are presented in Table 5.

**Figure 7:**
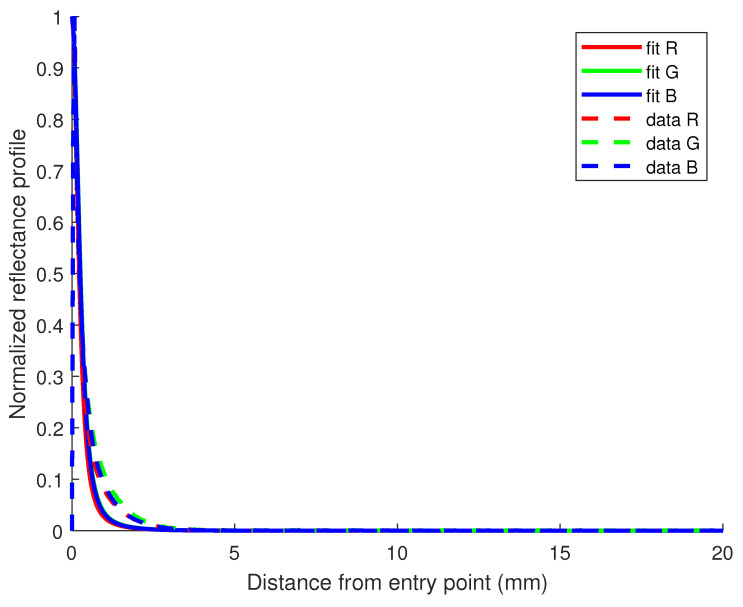
Reflectance profile for the paper 120 g/m${}^{2}$ in the Vertical dataset. $R^{2}$ values for the fit of R, G, and B channels are, respectively, 0.70, 0.72, and 0.73.

The first observation which can be made is that absorption values are largely lower than the reduced scattering values, which follows the assumption of highly diffuse materials. When comparing $\mu_{s}^{\prime }$ values between channels, one can note the red channel values are larger than the blue, which in turn are larger than the green. This could be interpreted as paper having a higher scattering capacity for the red wavelength over the blue and the green. One can also judge the quality of the estimates by using them with the diffusion model from Equation (1) and comparing the fit to the data. This is shown in Figure 7. The $R^{2}$ scores are correct with an average of 0.72. In the case of paper, it seems difficult to achieve a better result with this model and inversion method. Paper seems to be a highly scattering material, making it more challenging for the algorithm to properly estimate the absorption contribution, if any. This could be a reason to observe a difference between the fit and actual data at the lower part of the curve where it is the steepest.

#### 4.1.2. Horizontal Dataset Results

Similarly to the Vertical configuration, the absorption, and reduced scattering coefficients of white paper in the Horizontal configuration were computed and are registered in Table 6. The Horizontal configuration corresponds to the case for which the sensor is aligned with the orientation of paper sheets. Surprisingly, one can observe similar results for both $\mu_{a}$ and $\mu_{s}^{\prime }$ values with respect to the Vertical dataset. Indeed, the reduced scattering values have the same tendency regarding their behavior towards the wavelengths of the light source used. Values for the red channel are higher than the blue values and then the green values.

**Figure 8:**
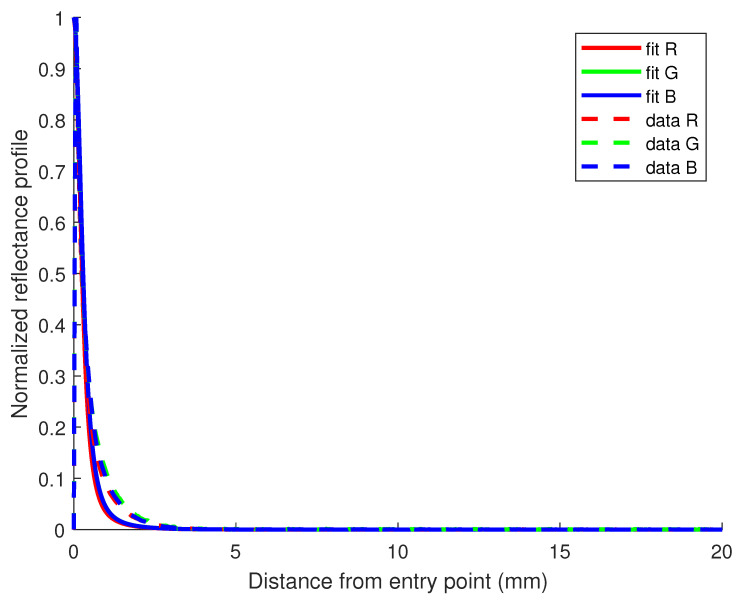
Reflectance profile for the paper 80 g/m${}^{2}$ in the Horizontal dataset. $R^{2}$ values for the fit of R, G, and B channels are, respectively, 0.73, 0.75, and 0.75.

The quality of these estimates can also be evaluated by using them to fit the diffusion model and compare it to the data, as shown on Figure 8. Similar to the Vertical dataset, the $R^{2}$ scores are a bit higher for this type of paper, but the same challenge appears when considering the steepest part of the curve. Although the diffusion model seems adequate for the configuration chosen, it might generate some underfitting for this data.

#### 4.1.3. Differences in Estimates between the Two Datasets

The second class of highly diffuse material we tested is white paper substrate. As previously mentioned, we experimented on two types of configurations for the paper and it resulted in various values of $\mu_{a}$ and $\mu_{s}^{\prime }$ for each paper substrate of different weight. Table 7 displays the computed relative difference of the reduced scattering coefficient for paper between the two configurations when taking the Vertical dataset as a reference. All of the relative differences are below 5% in absolute value. One can thus conclude the orientation of the paper substrate may not play a significant role for the scattering of the light when considering the diffusion approximation.

#### 4.1.4. Correlation with the Whiteness Index

In order to study the evolution of the estimates of absorption and reduced scattering coefficient between the different substrates, a physical index was chosen. The first choice was set on the paper’s weight since it naturally differentiates our substrates from each other, but we were hardly able to find relevant results. Thus we can assume that neither absorption nor scattering properties of paper are related to the weight of paper sheets. As the substrates are all white coloured, we oriented our choice of a physical index towards the Whiteness Index (WI). Several definitions can be found in the literature, and we selected two of them. The first WI selected follows the scale of CIE and is defined by

(11) $$WI=Y+800\left(x_{n}-x\right)+1700\left(y_{n}-y\right)$$

with $\left(x_{n},y_{n}\right)$ being coordinates which vary with the illuminant and the observer angle considered to measure the XYZ tristimuli of the material. The second WI chosen is defined in ASTM E313 of 1998 by

(12) WI=3.388Z−3Y

Both definitions are equivalent and for each, Y and Z can be deduced from the knowledge of (x,y,z) tristimuli. Thus, we used an EyeOne©Pro 3 spectrophotometer to measure the colour components of our substrates in XYZ space under the illuminant D50 and with an observer angle of 2°. Then values for $\left(x_{n},y_{n}\right)$ in Equation (11) are $\left(x_{n},y_{n}\right)=\left(0.3457,0.3585\right)$. Table 8 shows the results of the computed whiteness indices for our paper substrates.

With the computed whiteness indices and the estimated values of absorption and reduced scattering coefficients, we generated linear regression lines for each colour channel and each definition of WI. Results of this computation are presented in Table 9. A first general comment is to notice an increase in $R^{2}$ scores and a decrease in the associated *p*-values when going from the WI CIE definition to the WI E313. Even though the two indices are equivalent, one seems to give better results of linear correlation for $\mu_{a}$ and $\mu_{s}^{\prime }$ values. One can notice the $R^{2}$ scores and associated *p*-values for the red channel are good across the two datasets for each of the optical properties and whiteness indices. Regarding the scores of the green channel for the two WI, they are low, and the fact that *p*-values are high tends to mean the linear correlation between whiteness index and absorption or scattering properties of paper is insignificant for this specific wavelength. The results of the blue channel are mixed. The scores for $\mu_{a}$ of the Horizontal dataset are low for both WI, but the others for $\left(\mu_{a},\mu_{s}^{\prime }\right)$ of the Vertical dataset and $\mu_{s}^{\prime }$ for the Horizontal dataset are high, and this seems to show the existence of a linear correlation with the whiteness indices. Overall, these results clearly indicate a linear relationship between the whiteness indices and the absorption or scattering properties of paper substrates, but it varies with the wavelength considered.

### 4.2. Optical Properties of White Paint Mixed with Water

Another diffuse material we used for testing our inversion method is tainted water mixed with white acrylic paint. By increasing the concentration of pigments in the sample between each acquisition, we are able to monitor the evolution of absorption and reduced scattering coefficients of the mixture depending on the amount of paint dropped in it. To perform the acquisitions, we followed a similar protocol as the one utilized for milk measurements, as in both cases we are dealing with liquid materials. Thus, we filled out the same cylindrical transparent glass with tap water up until the height of 13.5 cm (out of a maximum of 16 cm for a diameter of 9 cm). It corresponds to an approximate volume of 850 milliliters. Then we added the drops of white acrylic paint and mixed them with water until reaching homogeneity before performing the acquisition. Due to the high volume of water, we started off the mixture by adding 2 drops of white paint, then mixed it and measured the reflectance profile with the TLS device. The incremental number of drops between each acquisition is 2 drops of white paint.

#### 4.2.1. Results

Results of the inversion method applied to the reflectance profiles are displayed in Table 10. $\mu_{s}^{\prime }$ estimated values are larger than $\mu_{a}$ values, which is expected from a diffuse material with high scattering properties. However, one may notice that absorption values for the first samples with 2 and 4 drops of white paint are extremely low, to the point their absorptive properties could be nullified. Due to the high initial volume of water in the glass, these concentrations of paint may not have been enough to change the absorption properties of the resulted mixture. The mixture was not diffuse enough, which could be visually checked, and thus some absorptive properties of the water may have stayed dominant. As a consequence, we chose to keep these points in the dataset for the results to be displayed and discussed, but we discard them for studying the correlation between the concentration of white paint and the estimated optical properties.

#### 4.2.2. Correlation with the Concentration of White Pigments

For this material, we wanted to check if a correlation could be found between absorption or scattering properties and the concentration of white pigments in the mixture. Since additional pigments in the mixture would increase the white perception and most likely its reflective properties, there might be a relation with the propagation of light in the material.

**Figure 9:**
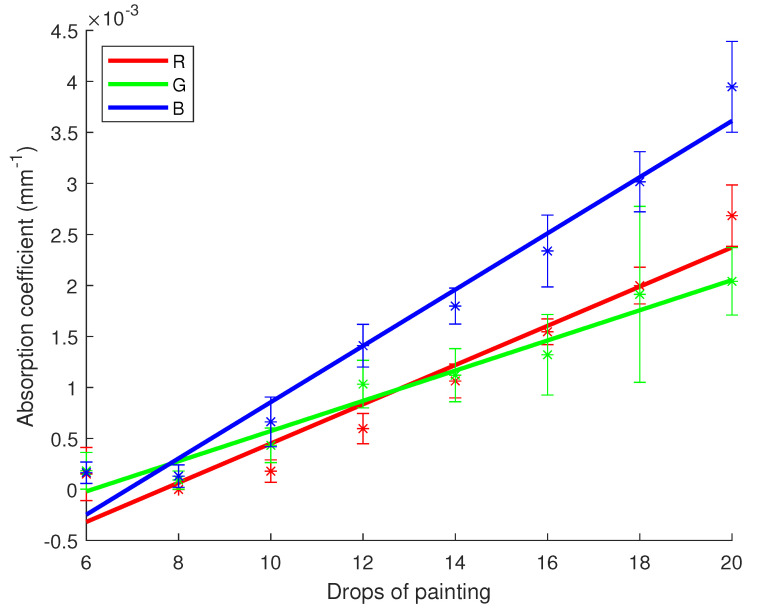
Linear regression for the absorption coefficient of white paint versus the drops number with 95% confidence intervals. Star symbols (*) represent the value of the coefficient with the uncertainty bar.

**Figure 10:**
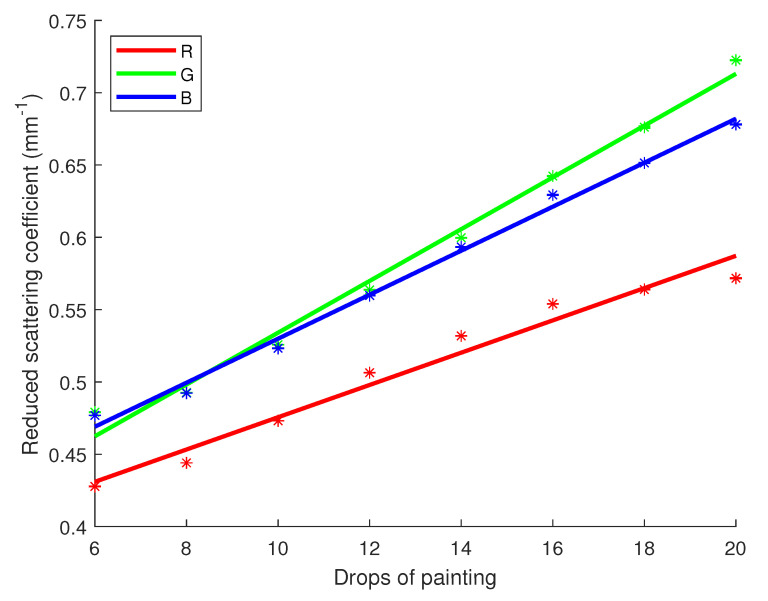
Linear regression for the reduced scattering coefficient of white paint versus the drops number with 95% confidence intervals. Star symbols (*) represent the value of the coefficient with the uncertainty bar.

$R^{2}$ scores and *p*-values for $\mu_{a}$ and $\mu_{s}^{\prime }$ regarding R, G and B channels are registered in Table 11. On the other hand, Figure 9 and Figure 10 show the linear regression lines respectively for $\mu_{a}$ and $\mu_{s}^{\prime }$. As one can note with the numbers of Table 11, a strong linear correlation has been identified for both optical properties depending on the concentration of white pigments. $R^{2}$ scores are in the range of 0.93 to 1 while *p*-values are very low, making it more possible for a linear correlation to exist. On Figure 9, one can notice the regression lines are going through their respective error bars. This is not the case in Figure 10, although the error bars are smaller due to less uncertainty and the points are quite aligned with the regression lines.

Therefore, it can be concluded a linear correlation exists between the concentration of white pigments and absorption or scattering coefficients. Even if our method of estimating the concentration of white pigments is as imprecise as just counting drops, one could study the way white paint on artworks absorbs or scatters light, and thus determine the initial concentration added to the mixture. Although in this case, the paint would be dry, which could affect the estimated coefficients.

## 5. Conclusions

We reviewed material appearance and methods to measure optical properties of the material. We used a spatially resolved measurement method with the Dia-Stron©TLS850 to obtain reflectance profiles of highly diffuse materials. Based on these data, we designed and applied a nonlinear inversion algorithm to fit the diffusion model by splitting the reflectance curve in two parts to solve the ill-posed problem when only having a single measurement. This resulted in estimates of absorption and reduced scattering coefficients of the material. The comparison of estimates for milk samples with values from the literature allowed us to validate our method. The values of absorption coefficients retrieved vary between 1 × 10${}^{-3}$ and 8 × 10${}^{-3}$ mm${}^{-1}$, whilst the values of the scattering coefficients obtained from our method are between 3 and 8 mm${}^{-1}$, depending on the percentage of fat in the milk sample, and under the assumption of the anisotropy factor g>0.8. When comparing to the current literature, values for the scattering coefficient usually vary between 1 and 5 mm${}^{-1}$, while absorption coefficient values are between 1 × 10${}^{-1}$ and 1 mm${}^{-1}$, depending on the studies performed [7,33,36]. Although there are some variations for the range of absorption coefficients, the range of scattering values estimated overlaps the one from the literature. These results were also supported by finding a linear correlation between optical properties and the fat content of milk, as we obtained a good fitting given by $R^{2}$ scores over 0.94 with low *p*-values, thus demonstrating the validity of our designed method. Furthermore, we investigated paper and white paint materials. We observed a linear correlation between the concentration of paint pigments and optical coefficients. In the case of paper, it was more challenging. As we have mentioned, the paper structure is anisotropic and we would thus require more measurements at different angles, then add them in the model to consider the anisotropy structure and to obtain a good estimates of the properties. A future work could be to invent a new measuring device with a central light source surrounded by CCD sensors in different directions to be used for anisotropic materials. Therefore, we can conclude our measurement method performs well on highly diffuse and isotropic materials, but it should work with diffuse materials too as long as the samples considered are large enough to use the semi-infinite assumption and the measuring instrument is adapted.

However, challenges remain for this measurement method and class of materials. We mainly used relative quantities but it should be possible to explore the field of measuring objective quantities. Furthermore, the model could be adapted to directly estimate the scattering coefficient of a material instead of the reduced scattering, then requiring additional knowledge on the anisotropy factor. The method could even be refined to provide more precise values regarding the absorption coefficient. Eventually, this method could be compared to other methods (Monte Carlo simulations, machine learning based methods) used in the literature to measure optical properties of diffuse materials. Generally, this work has validated the method, while there are possibilities for improvements. Thus, it allows us to continue to study materials with little stability or a fast evolution in situ. Potential contexts or materials to be considered are the process of 3D printed white objects, dairy food manufacturing, or the evolution of snow for climate monitoring.

## Figures and Tables

**Table 1:** Estimated optical properties for the Q-dataset.

Fat Content	$\mu_{a}$ (mm${}^{-1}$)			$\mu_{s}^{\prime }$ (mm${}^{-1}$)		
	R	G	B	R	G	B
**0.1%**	3.2 × 10${}^{-9}$	2.4 × 10${}^{-3}$	1.0 × 10${}^{-2}$	0.62	0.74	0.81
**0.5%**	1.4 × 10${}^{-3}$	3.7 × 10${}^{-3}$	1.2 × 10${}^{-2}$	0.62	0.75	0.84
**1%**	3.0 × 10${}^{-3}$	7.8 × 10${}^{-3}$	1.6 × 10${}^{-2}$	0.69	0.84	0.95
**4%**	5.3 × 10${}^{-3}$	1.5 × 10${}^{-2}$	3.6 × 10${}^{-2}$	1.05	1.35	1.52

**Table 2:** Estimated optical properties for the mix-dataset.

Fat Content	$\mu_{a}$ (mm${}^{-1}$)			$\mu_{s}^{\prime }$ (mm${}^{-1}$)		
	R	G	B	R	G	B
**0.1%**	1.7 × 10${}^{-5}$	3.4 × 10${}^{-3}$	8.6 × 10${}^{-3}$	0.60	0.70	0.78
**0.5%**	1.2 × 10${}^{-3}$	4.0 × 10${}^{-3}$	1.3 × 10${}^{-2}$	0.61	0.75	0.83
**0.7%**	1.9 × 10${}^{-3}$	5.0 × 10${}^{-3}$	1.4 × 10${}^{-2}$	0.67	0.82	0.91
**1%**	3.3 × 10${}^{-3}$	6.6 × 10${}^{-3}$	1.9 × 10${}^{-2}$	0.68	0.84	0.92
**1.2%**	4.3 × 10${}^{-3}$	5.8 × 10${}^{-3}$	1.9 × 10${}^{-2}$	0.71	0.90	0.98
**3.5%**	5.1 × 10${}^{-3}$	1.2 × 10${}^{-2}$	2.7 × 10${}^{-2}$	0.92	1.26	1.40
**4%**	5.3 × 10${}^{-3}$	1.1 × 10${}^{-2}$	3.5 × 10${}^{-2}$	0.98	1.36	1.51

**Table 3:** Results of statistical studies for the milk mix-dataset.

Channel		R	G	B
$\mu_{a}$	$R^{2}$	0.86	0.94	0.95
	*p*-value	2.8 × 10${}^{-3}$	3.5 × 10${}^{-4}$	2.2 × 10${}^{-4}$
$\mu_{s}^{\prime }$	$R^{2}$	0.98	1.00	1.00
	*p*-value	1.6 × 10${}^{-6}$	7.1 × 10${}^{-8}$	3.2 × 10${}^{-8}$

**Table 4:** Relative difference of estimated optical values between the two datasets. The Q-dataset is considered as reference value.

Fat Content	Relative Difference of $\mu_{a}$			Relative Difference of $\mu_{s}^{\prime }$		
	R	G	B	R	G	B
**0.1%**	46.9%	−41.7%	14%	3.2%	5.4%	3.7%
**0.5%**	14.3%	−8.1%	−8.3%	1.6%	0%	1.2%
**1%**	−10%	15.4%	−18.8%	1.4%	0%	3.2%
**4%**	0%	26.7%	2.8%	6.7%	−0.7%	0.7%

**Table 5:** Estimated optical properties for vertical dataset.

Weight (g/m${}^{2}$)	$\mu_{a}$ (mm${}^{-1}$)			$\mu_{s}^{\prime }$ (mm${}^{-1}$)		
	R	G	B	R	G	B
**80**	6.62 × 10${}^{-2}$	8.51 × 10${}^{-2}$	8.38 × 10${}^{-2}$	3.13	2.61	2.69
**100**	4.83 × 10${}^{-2}$	7.96 × 10${}^{-2}$	7.65 × 10${}^{-2}$	3.45	2.86	2.91
**120**	5.70 × 10${}^{-2}$	7.75 × 10${}^{-2}$	8.21 × 10${}^{-2}$	3.37	2.81	2.86
**160**	3.86 × 10${}^{-2}$	5.41 × 10${}^{-2}$	5.44 × 10${}^{-2}$	3.59	3.08	3.19
**200**	3.51 × 10${}^{-2}$	5.90 × 10${}^{-2}$	5.27 × 10${}^{-2}$	3.63	3.00	3.16
**250**	4.20 × 10${}^{-2}$	7.14 × 10${}^{-2}$	5.45 × 10${}^{-2}$	3.51	2.77	3.09

**Table 6:** Estimated optical properties for horizontal dataset.

Weight (g/m${}^{2}$)	$\mu_{a}$ (mm${}^{-1}$)			$\mu_{s}^{\prime }$ (mm${}^{-1}$)		
	R	G	B	R	G	B
**80**	6.34 × 10${}^{-2}$	8.06 × 10${}^{-2}$	8.00 × 10${}^{-2}$	2.97	2.55	2.56
**100**	5.07 × 10${}^{-2}$	8.28 × 10${}^{-2}$	7.76 × 10${}^{-2}$	3.33	2.80	2.80
**120**	5.43 × 10${}^{-2}$	6.41 × 10${}^{-2}$	8.15 × 10${}^{-2}$	3.29	2.94	2.88
**160**	4.45 × 10${}^{-2}$	5.67 × 10${}^{-2}$	5.97 × 10${}^{-2}$	3.43	3.04	3.08
**200**	4.33 × 10${}^{-2}$	6.77 × 10${}^{-2}$	6.83 × 10${}^{-2}$	3.48	2.97	3.04
**250**	4.26 × 10${}^{-2}$	7.07 × 10${}^{-2}$	7.05 × 10${}^{-2}$	3.52	2.87	3.07

**Table 7:** Relative difference of reduced scattering values between the Vertical and Horizontal datasets. The Vertical dataset is taken as a reference.

Weight (g/m${}^{2}$)	Relative Difference of $\mu_{s}^{\prime }$		
	R	G	B
**80**	5.1%	2.3%	4.8%
**100**	3.5%	2.1%	3.8%
**120**	2.4%	−4.6%	−0.7%
**160**	4.5%	1.3%	3.4%
**200**	4.1%	1.0%	3.8%
**250**	−0.3%	−3.6%	0.6%

**Table 8:** Whiteness indices of paper substrates.

Weight (g/m${}^{2}$)	80	100	120	160	200	250
WI CIE	133.4	135.1	137.4	140.1	141.2	142.6
WI E313	23.06	25.92	29.13	32.32	34.02	34.45

**Table 9:** Results of statistical studies for the paper datasets.

		WI CIE			WI E313		
		R	G	B	R	G	B
$\mu_{a}$ (ver)	$R^{2}$	0.72	0.43	0.79	0.76	0.49	0.77
	*p*-value	0.03	0.16	0.02	0.02	0.12	0.02
$\mu_{a}$ (hor)	$R^{2}$	0.84	0.58	0.24	0.88	0.63	0.29
	*p*-value	0.01	0.08	0.33	0.006	0.06	0.27
$\mu_{s}^{\prime }$ (ver)	$R^{2}$	0.67	0.30	0.79	0.75	0.39	0.84
	*p*-value	0.05	0.26	0.02	0.03	0.18	0.01
$\mu_{s}^{\prime }$ (hor)	$R^{2}$	0.78	0.58	0.86	0.82	0.69	0.91
	*p*-value	0.02	0.08	0.007	0.01	0.04	0.003

**Table 10:** Estimated optical properties for the white paint dataset.

Drops	$\mu_{a}$ (mm${}^{-1}$)			$\mu_{s}^{\prime }$ (mm${}^{-1}$)		
	R	G	B	R	G	B
**2**	6.1 × 10${}^{-12}$	2.8 × 10${}^{-11}$	1.5 × 10${}^{-11}$	0.45	0.46	0.50
**4**	2.0 × 10${}^{-12}$	1.4 × 10${}^{-13}$	1.4 × 10${}^{-11}$	0.40	0.43	0.44
**6**	2.3 × 10${}^{-5}$	6.4 × 10${}^{-5}$	9.3 × 10${}^{-5}$	0.43	0.48	0.48
**8**	1.1 × 10${}^{-10}$	3.8 × 10${}^{-5}$	6.5 × 10${}^{-5}$	0.44	0.49	0.49
**10**	1.3 × 10${}^{-4}$	3.5 × 10${}^{-4}$	5.5 × 10${}^{-4}$	0.47	0.53	0.52
**12**	5.7 × 10${}^{-4}$	1.0 × 10${}^{-3}$	1.4 × 10${}^{-3}$	0.51	0.56	0.56
**14**	1.0 × 10${}^{-3}$	1.1 × 10${}^{-3}$	1.8 × 10${}^{-3}$	0.53	0.60	0.59
**16**	1.5 × 10${}^{-3}$	1.1 × 10${}^{-3}$	2.3 × 10${}^{-3}$	0.55	0.64	0.63
**18**	2.0 × 10${}^{-3}$	1.7 × 10${}^{-3}$	3.0 × 10${}^{-3}$	0.56	0.68	0.65
**20**	2.7 × 10${}^{-3}$	2.0 × 10${}^{-3}$	3.9 × 10${}^{-3}$	0.57	0.72	0.68

**Table 11:** Results of statistical studies for the white paint dataset.

Channel		R	G	B
$\mu_{a}$	$R^{2}$	0.93	0.96	0.97
	*p*-value	1.2 × 10${}^{-4}$	2.6 × 10${}^{-5}$	9.2 × 10${}^{-6}$
$\mu_{s}^{\prime }$	$R^{2}$	0.97	0.99	0.99
	*p*-value	9.8 × 10${}^{-6}$	3.1 × 10${}^{-7}$	7.7 × 10${}^{-8}$

## Data Availability

The code written in Matlab© is available on the Github page of our department at the following link https://github.com/Colourlab-NTNU, accessed on 3 July 2023.

## References

[1] Sun C.C., Chien W.T., Moreno I., Hsieh C.T., Lin M.C., Hsiao S.L., Lee X.H. (2010). Calculating model of light transmission efficiency of diffusers attached to a lighting cavity. Opt. Express.

[2] Leyre S., Leloup F., Audenaert J., Durinck G., Hofkens J., Deconinck G., Hanselaer P. (2013). Determination of the bulk scattering parameters of diffusing materials. Appl. Opt..

[3] Cheong W., Prahl S., Welch A.J. (1990). A review of the optical properties of biological tissues. IEEE J. Quantum Electron..

[4] Gobin L., Blanchot L., Saint-Jalmes H. (1999). Integrating the digitized backscattered image to measure absorption and reduced-scattering coefficients in vivo. Appl. Opt..

[5] Hyde D.E., Farrell T.J., Patterson M.S., Wilson B.C. (2001). A diffusion theory model of spatially resolved fluorescence fromdepth-dependent fluorophore concentrations. Phys. Med. Biol..

[6] Zhang R., Verkruysse W., Choi B., Viator J.A., Jung B., Svaasand L.O., Aguilar G., Nelson J.S. (2005). Determination of human skin optical properties from spectrophotometric measurements based on optimization by genetic algorithms. J. Biomed. Opt..

[7] Qin J., Lu R. (2007). Measurement of the Absorption and Scattering Properties of Turbid Liquid Foods Using Hyperspectral Imaging. Appl. Spectrosc..

[8] Qin J., Lu R. (2008). Measurement of the optical properties of fruits and vegetables using spatially resolved hyperspectral diffuse reflectance imaging technique. Postharvest Biol. Technol..

[9] Hu D., Fu X., Wang A., Ying Y. (2015). Measurement Methods for Optical Absorption and Scattering Properties of Fruits and Vegetables. Trans. ASABE.

[10] Guarnera G.C., Ghosh A., Hall I., Glencross M., Guarnera D. Material Capture and Representation with Applications in Virtual Reality. Proceedings of the SIGGRAPH’17: Special Interest Group on Computer Graphics and Interactive Techniques Conference.

[11] Tong X., Wang J., Lin S., Guo B., Shum H.Y. (2005). Modeling and Rendering of Quasi-Homogeneous Materials. ACM Trans. Graph..

[12] (2022). Standard Terminology of Appearance.

[13] Nicodemus F.E., Richmond J.C., Hsia J.J., Ginsberg I.W., Limperis T. (1977). Geometrical Considerations and Nomenclature for Reflectance.

[14] Doornbos R., Lang R., Aalders M., Cross F., Sterenborg H. (1999). The determination of in vivo human tissue optical properties and absolute chromophore concentrations using spatially resolved steady-state diffuse reflectance spectroscopy. Phys. Med. Biol..

[15] Igarashi T., Nishino K., Nayar S.K. (2007). The Appearance of Human Skin: A Survey. Found. Trends. Comput. Graph. Vis..

[16] Farrell T.J., Patterson M.S., Wilson B. (1992). A diffusion theory model of spatially resolved, steady-state diffuse reflectance for the noninvasive determination of tissue optical properties in vivo. Med. Phys..

[17] Frisvad J.R., Jensen S.A., Madsen J.S., Correia A., Yang L., Gregersen S.K.S., Meuret Y., Hansen P.E. (2020). Survey of Models for Acquiring the Optical Properties of Translucent Materials. Comput. Graph. Forum.

[18] Kubelka P., Munk F. (1931). Ein Beitrag zur Optik der Farbanstriche. Z. Tech. Phys..

[19] Maheu B., Gouesbet G. (1986). Four-flux models to solve the scattering transfer equation: Special cases. Appl. Opt..

[20] Prahl S.A., van Gemert M.J.C., Welch A.J. (1993). Determining the optical properties of turbid media by using the adding-doubling method. Appl. Opt..

[21] Bashkatov A.N., Genina E.A., Tuchin V.V. (2011). Optical Properties of Skin, Subcutaneous, and Muscle tissues: A Review. J. Innov. Opt. Health Sci..

[22] Tuchin V. (2015). Tissue Optics: Light Scattering Methods and Instruments for Medical Diagnostics.

[23] Munoz A., Masia B., Tolosa A., Gutierrez D. (2009). Single-image appearance acquisition using genetic algorithms. Proc. Comput. Graph. Vis. Comput. Vis. Image Process..

[24] Munoz A., Echevarria J.I., Seron F.J., Lopez-Moreno J., Glencross M., Gutierrez D. (2011). BSSRDF Estimation from Single Images. Comput. Graph. Forum.

[25] Peers P., vom Berge K., Matusik W., Ramamoorthi R., Lawrence J., Rusinkiewicz S., Dutré P. (2006). A Compact Factored Representation of Heterogeneous Subsurface Scattering. ACM Trans. Graph..

[26] Kienle A., Lilge L., Patterson M.S., Hibst R., Steiner R., Wilson B.C. (1996). Spatially resolved absolute diffuse reflectance measurements for noninvasive determination of the optical scattering and absorption coefficients of biological tissue. Appl. Opt..

[27] Kienle A., Patterson M.S. (1996). Determination of the optical properties of turbid media from a single Monte Carlo simulation. Phys. Med. Biol..

[28] Kienle A., Patterson M.S. (1997). Determination of the optical properties of semi-infinite turbid media from frequency-domain reflectance close to the source. Phys. Med. Biol..

[29] Stam J., Patrick M., Purgathofer W. (1995). Multiple scattering as a diffusion process. Rendering Techniques’ 95, Proceedings of the Eurographics Workshop, Dublin, Ireland, 12–14 June 1995.

[30] Jensen H.W., Marschner S.R., Levoy M., Hanrahan P. A Practical Model for Subsurface Light Transport. Proceedings of the 28th Annual Conference on Computer Graphics and Interactive Techniques, Association for Computing Machinery.

[31] Donner C., Jensen H.W. (2005). Light Diffusion in Multi-Layered Translucent Materials. ACM Trans. Graph..

[32] Nichols M.G., Hull E.L., Foster T.H. (1997). Design and testing of a white-light, steady-state diffuse reflectance spectrometer for determination of optical properties of highly scattering systems. Appl. Opt..

[33] Stocker S., Foschum F., Krauter P., Bergmann F., Hohmann A., Happ C.S., Kienle A. (2017). Broadband Optical Properties of Milk. Appl. Spectrosc..

[34] Aernouts B., Van Beers R., Watté R., Huybrechts T., Jordens J., Vermeulen D., Van Gerven T., Lammertyn J., Saeys W. (2015). Effect of ultrasonic homogenization on the Vis/NIR bulk optical properties of milk. Colloid Surf. B Biointerfaces.

[35] Wabnitz H., Rinneberg H. (1997). Imaging in turbid media by photon density waves: Spatial resolution and scaling relations. Appl. Opt..

[36] Abildgaard O.H.A., Kamran F., Dahl A.B., Skytte J.L., Nielsen F.D., Thomsen C.L., Andersen P.E., Larsen R., Frisvad J.R. (2015). Non-Invasive Assessment of Dairy Products Using Spatially Resolved Diffuse Reflectance Spectroscopy. Appl. Spectrosc..

[37] Bahadi M., Ismail A.A., Vasseur E. (2021). Fourier Transform Infrared Spectroscopy as a Tool to Study Milk Composition Changes in Dairy Cows Attributed to Housing Modifications to Improve Animal Welfare. Foods.

[38] Blinov A., Siddiqui S., Blinova A., Khramtsov A., Oboturova N., Nagdalian A., Simonov A., Ibrahim S. (2022). Analysis of the dispersed composition of milk using photon correlation spectroscopy. J. Food Compos. Anal..

[39] Farrell T.J., Wilson B.C., Patterson M.S. (1992). The use of a neural network to determine tissue optical properties from spatially resolved diffuse reflectance measurements. Phys. Med. Biol..

[40] Bevilacqua F., Depeursinge C. (1999). Monte Carlo study of diffuse reflectance at source–detector separations close to one transport mean free path. J. Opt. Soc. Am. A.

[41] Fabritius T., Saarela J., Myllyla R. (2006). Determination of the refractive index of paper with clearing agents. International Conference on Lasers, Applications, and Technologies 2005: High-Power Lasers and Applications.

